# Current nutrition promotion, beliefs and barriers among cancer nurses in Australia and New Zealand

**DOI:** 10.7717/peerj.1396

**Published:** 2015-11-10

**Authors:** Petra G. Puhringer, Alicia Olsen, Mike Climstein, Sally Sargeant, Lynnette M. Jones, Justin W.L. Keogh

**Affiliations:** 1 Department of Neurology, Medical University of Vienna, Vienna, Austria; 2 Faculty of Health Sciences and Medicine, Bond University, Robina, Queensland, Australia; 3 Exercise, Health and Performance Faculty Research Group, University of Sydney, Sydney, New South Wales, Australia; 4 School of Physical Education, Sport & Exercise Sciences, University of Otago, Dunedin, Otago, New Zealand; 5 Human Potential Centre, AUT University, Auckland, New Zealand; 6 Cluster for Health Improvement, Faculty of Science, Health, Education and Engineering, University of the Sunshine Coast, Sippy Downs, Australia

**Keywords:** Cancer nurses, Health professional, Nutrition, Cancer patients, Healthy eating, Oncology, Health promotion

## Abstract

**Rationale.** Many cancer patients and survivors do not meet nutritional and physical activity guidelines, thus healthier eating and greater levels of physical activity could have considerable benefits for these individuals. While research has investigated cancer survivors’ perspective on their challenges in meeting the nutrition and physical guidelines, little research has examined how health professionals may assist their patients meet these guidelines. Cancer nurses are ideally placed to promote healthy behaviours to their patients, especially if access to dieticians or dietary resources is limited. However, little is known about cancer nurses’ healthy eating promotion practices to their patients. The primary aim of this study was to examine current healthy eating promotion practices, beliefs and barriers of cancer nurses in Australia and New Zealand. A secondary aim was to gain insight into whether these practices, beliefs and barriers were influenced by the nurses’ hospital or years of work experience.

**Patients and Methods.** An online questionnaire was used to obtain data. Sub-group cancer nurse comparisons were performed on hospital location (metropolitan vs regional and rural) and years of experience (<25 or ≥25 years) using ANOVA and chi square analysis for continuous and categorical data respectively.

**Results.** A total of 123 Australasian cancer nurses responded to the survey. Cancer nurses believed they were often the major provider of nutritional advice to their cancer patients (32.5%), a value marginally less than dieticians (35.9%) but substantially higher than oncologists (3.3%). The majority promoted healthy eating prior (62.6%), during (74.8%) and post treatment (64.2%). Most cancer nurses felt that healthy eating had positive effects on the cancer patients’ quality of life (85.4%), weight management (82.9%), mental health (80.5%), activities of daily living (79.7%) and risk of other chronic diseases (79.7%), although only 75.5% agreed or strongly agreed that this is due to a strong evidence base. Lack of time (25.8%), adequate support structures (17.3%) nutrition expertise (12.2%) were cited by the cancer nurses as the most common barriers to promoting healthy eating to their patients. Comparisons based on their hospital location and years of experience, revealed very few significant differences, indicating that cancer nurses’ healthy eating promotion practices, beliefs and barriers were largely unaffected by hospital location or years of experience.

**Conclusion.** Australasian cancer nurses have favourable attitudes towards promoting healthy eating to their cancer patients across multiple treatment stages and believe that healthy eating has many benefits for their patients. Unfortunately, several barriers to healthy eating promotion were reported. If these barriers can be overcome, nurses may be able to work more effectively with dieticians to improve the outcomes for cancer patients.

## Introduction

Cancer rates are rising in many countries, with Australian 2010 data indicating that within an overall population of 22 million people, there were 116,580 new cases of cancer diagnosed that year (Australian Institute of Health and Welfare, 2014). This very high number of new cancer cases in Australia is consistent with many other countries and may reflect many factors including an increased lifespan of the population (Brown, Lipscomb & Snyder, 2001; Li, 2010) and improved detection (Newton & Galvão, 2008). In addition, several modifiable risk factors appear to contribute to these higher cancer rates, including insufficient levels of physical activity, poor dietary choices and other unhealthy lifestyle choices, such as smoking (Boyle et al., 2012; De Stefani et al., 2013; Moorman et al., 2011).

Earlier detection of many cancers and advancements in surgical techniques, radiation therapy, chemotherapy and hormonal therapies allow many more individuals with cancer to survive longer post-diagnosis (Etzioni et al., 2008). Survival rates of 88% and 85% for prostate and breast cancer respectively, indicate that 53,296 Australian men diagnosed with prostate cancer and 53,051 Australian women diagnosed with breast cancer are still alive 5 years post-diagnosis (Australian Institute of Health and Welfare & Australasian Association of Cancer Registries, 2010). While survival rates are high, cancer treatments result in many short- and long-term side-effects that seriously affect the quality of life (QoL) and overall health and wellbeing of these individuals (Mazzotti et al., 2012). Many of these treatments may contribute to cancer fatigue, with hormonal therapies also predisposing these individuals to unhealthy changes in body composition such as cachexia or sarcopenic obesity (Galvao et al., 2008; Young et al., 2014). Cancer cachexia is described as a multifactorial syndrome involving the continual loss of skeletal muscle mass (with or without loss of fat mass) that is not reversible by conventional nutritional support (Fearon et al., 2011). Sarcopenic obesity is a condition characterised by significant reductions in muscle and bone mass and increases in fat mass (Berger, Gerber & Mayer, 2012; Bylow et al., 2007; Genton et al., 2006). Hormonal therapies (such as androgen deprivation therapy) and chemotherapy can lead to similar significant declines in body composition that predispose cancer patients to cachexia or sarcopenic obesity (Galvao et al., 2008; Young et al., 2014). Moreover, these alterations in body weight and composition may be associated with reduced functional status, the development of co-morbidities such as osteoporosis, fall-related fractures, and cardio-metabolic syndrome (Bundred, 2012; Kintzel et al., 2008; Oefelein et al., 2002; Young et al., 2014), increased rates of chemotherapy toxicity (Azim et al., 2011; Prado et al., 2008) and may be linked to decreased survival rates (Sparano et al., 2012). Many cancer survivors also live with numerous additional symptoms including poor sleep, urinary and sexual dysfunction, and negative body image that may further impact aspects of their QoL and their ability to perform self-care, work, and leisure activities (Baker et al., 2005; Flynn et al., 2011; Keogh et al., 2013; Ottenbacher et al., 2013). Such results suggest that improved treatments may allow cancer survivors to live longer, but this may be achieved with substantial losses of QoL. Improvements in usual care practices are therefore needed to increase the overall health and QoL post-diagnosis for all cancer populations.

We would argue that healthy behaviours like physical activity, adequate nutrition, weight management and smoking cessation have considerable health benefits for cancer survivors and should be strongly considered to become more routinely integrated into usual care practices (Demark-Wahnefried et al., 2015). Meta-analyses and systematic reviews indicate that improved dietary choices are an important component of enhanced health and QoL among cancer patients including weight maintenance as well as reduced levels of diabetes and obesity, osteoporosis, and potentially cancer recurrence (Langius et al., 2013; Millar & Davison, 2012; Mokdad et al., 2003).

Unfortunately, a recent review focussing on cancer survivors with different cancer types in Canada and the United States indicates that too few cancer patients engage in healthy eating and sufficient physical activity for health benefits (Bellizzi et al., 2005; Blanchard, Courneya & Stein, 2008; Courneya, Katzmarzyk & Bacon, 2008; Demark-Wahnefried et al., 2015). Adherence to specific dietary guidelines (i.e., five servings of fruits or vegetables a day, high intake of whole grains and a low intake of red and processed meats, refined grains and sugar) was practiced only by a minority of cancer survivors across six major cancer types, with between 14.8% to 19.1% consuming the recommended daily amount of fruits and vegetables, and between 29.6% to 47.3% engaging in the advised physical activity (Blanchard, Courneya & Stein, 2008). Poor health behaviours are an issue in a variety of cancer settings, especially where patients are at risk of cachexia or sarcopenic obesity. For those patients at risk of cachexia or sarcopenic obesity, a primary dietary aim is to obtain sufficiently balanced nutrition (particularly protein and overall calories) to maintain levels of muscle mass and perhaps to limit the gain in fat mass (Balstad et al., 2014; Chevalier & Farsijani, 2014).

Previous findings suggest that cancer survivors are more likely to increase their healthy behaviours if oncologists and/or the other health professionals in the oncology care team e.g., cancer nurses, actively promote lifestyle modification as a tertiary preventative strategy, preferably as soon as possible after initiation of the individual treatment plan (Jones et al., 2004; Jones & Demark-Wahnefried, 2006; Karvinen et al., 2012). One challenge facing health professionals (e.g., oncologists and cancer nurses) becoming more active in promoting healthy eating to their patients is that this is often not their primary area of training or scope of practice. However, when there are limited human resources, particularly dieticians/nutritionists, available to the cancer patients, an interdisciplinary approach should be considered to provide cancer survivors with general healthy eating guidelines. Nevertheless, oncologists and cancer nurses have several concerns when they feel responsible for promoting a healthy diet to their patients, ranging from the belief that diet wouldn’t impact the cancer outcome, or that the cancer survivor may interpret such discussions as laying the blame for their cancer diagnosis on their poor lifestyle choices (Williams et al., 2015).

A diagnosis of cancer may encourage patients to change their lifestyle habits, becoming more physically active, eating a better diet or quitting smoking (Anderson, Steele & Coyle, 2013; Demark-Wahnefried et al., 2000). Health professionals are in a position to actively promote and/or respond to questions about these behaviours, thereby assisting their patients to make achievable lifestyle changes (Velentzis et al., 2011). Such promotion has the potential to result in many health benefits. For example, in prostate cancer patients receiving androgen deprivation therapy who are often at risk of osteoporosis, the promotion of sufficient calcium and vitamin D intake has been shown to be effective in reducing bone loss (Davison, Wiens & Cushing, 2012).

Nurses have a key role in communication between cancer patients and the wider oncology care team (which often consists of various medical specialists and institutions) as they may have more frequent contact with their patients and relatively more time per consultation for counselling their patients on supportive care issues than oncologists (Leahy et al., 2012). However, there is no specific data providing information on how cancer nurses see their role in promoting healthy eating to their patients. Evidence from nurses in general practice (Van Dillen et al., 2014) and paediatric (Blake & Patterson, 2015) settings indicate that nurses saw themselves as important health professionals in relation to promoting healthy eating to their patients. Cancer nurses, however, may face a number of barriers affecting their ability to promote healthy behaviours to their patients (Karvinen et al., 2012). Lack of guidelines and lack of time was often cited as barriers (O’Hanlon & Kennedy, 2014) as well as the desire to minimise patients’ distress (Miles, Simon & Wardle, 2010) and the relative lack of access to evidence-based resources to provide to their patients (Blake & Patterson, 2015).

There is little international data on the current healthy eating promotion practices, beliefs and barriers of cancer nurses. It is also apparent that almost all healthy behaviour promotion by health professionals has focused on physical activity and been conducted in North America and Europe (Daley et al., 2008; Jones et al., 2005a; Karvinen et al., 2012; Kotronoulas, Papadopoulou & Patiraki, 2009; Williams et al., 2015), with virtually no research conducted in Australasia (Australia and New Zealand) (Spellman, Craike & Livingston, 2014). The current study aimed to address these limitations within the literature by gaining some insight into the current healthy eating promotion practices, beliefs and barriers of cancer nurses in Australia and New Zealand. A secondary goal was to gain preliminary insight into whether these practices and determinants were influenced by the location of the nurses’ hospitals (rural, regional and metropolitan) or years of work experience. It was hypothesised that cancer nurses would, in principle, support the promotion of healthy diets to their patients and believe that a healthy diet has many benefits, but that they would cite many barriers to the promotion of healthy eating to their patients.

## Methods

### Participants and procedures

Registered cancer nurses (RNs) who provide medical support to cancer patients in either Australia or New Zealand were invited to participate in our online survey. Australian cancer nurses were invited to participate via links posted on the Cancer Nurses Society of Australia (CNSA) website, while New Zealand cancer nurses were invited via an email from the Cancer Nurses Section of the New Zealand Nurses Organisation (NZNO). The web link or email the nurses received from their nursing organisations included a description of the research and an invitation to participate in the study via an electronic link to the online questionnaire. Additionally, any registered nurses providing health care to cancer patients were eligible to participate; these potential participants were recruited using social media (Twitter and Facebook).

### Ethics approval

Institutional approval for this study was obtained from the Bond University Human Review Ethics committee (RO1651) and the University of Otago Human Ethics Committee (13/260) and organizational approval obtained from both the CNSA and NZNO. Ethics approval included permission to offer an incentive for participation, which consisted of twenty-four, $20 gift vouchers that were randomly allocated to participants who completed the survey. All participants provided informed consent electronically on the main survey page prior to accessing the online survey.

### Survey design and implementation

A cross-sectional, observational study was designed using an online, web-based questionnaire survey software (SurveyMonkey Inc., Palo Alto, California, USA). The survey questions consisted of array, single choice, multiple choice, list dropdown, numerical input and short answer free text. Filters and skip logic (where appropriate) were utilized to expedite completion of the survey. The questionnaire was initially trialled with 12 nurses, with only minor changes made to the terminology or layout prior to being made available online. The Australian survey was activated on October 2013 and closed on July 2014 and the New Zealand survey was activated on December 2013 and also closed July 2014.

### Study instrument

The online survey questionnaire was based upon two key theoretical frameworks within health behaviour research, namely the Theory of Planned Behaviour (TPB) and the Social Cognitive Theory (SCT). The components of the TPB comprise normative beliefs, perceived control and intentions (Azjen, 1985), whilst SCT places emphasis on thought process constructs that govern behaviour (Bandura, 1986). The survey questions were designed to reflect these constructs within the two theories, as well as drawing on other key studies within the literature that identified determinants of healthy living in cancer patients (Blanchard et al., 2002; Jones et al., 2005b). The constructs and factors of these theories could be applied to how patients perceive the opinion and advice of health professionals such as oncologists and cancer, in relation to the performance of healthy behaviours (Husebo et al., 2013; Jones et al., 2005b; Keogh et al., 2010; Short, James & Plotnikoff, 2013). The questionnaire used the guiding principles of TPB and SCT to assess cancer nurses’ practice towards nutrition promotion, attitudes towards beneficial effects of healthy eating in cancer patients and their perceived barriers for healthy eating promotion. The wider survey was divided into four major sections, these being demographics of the cancer nurses and three healthy living components which included cancer nurses’ beliefs about the importance of healthy eating, physical activity and smoking habits in the treatment process of cancer survivors. Questions about healthy eating, physical activity and smoking were incorporated to minimise response bias. In particular, this approach was done to minimise the potential for the nurses who did not promote a specific healthy behaviour e.g., healthy eating to decline the invitation to participate in the survey, only answer some of the questions in the survey or to feel pressured to give response(s) that was/were not consistent with their actual behaviour or beliefs. In this paper, only data relevant to the cancer nurses’ healthy eating promotion habits and beliefs towards their patients will be reported.

Demographics obtained from the cancer nurses included age, gender, professional qualifications, years practicing, practice type (public/private), hospital location (metropolitan, regional, rural) and specialisation (cancer or tumour group). Assessment of cancer nurses’ lifestyle habits consisted of single-choice questions. Items included their current smoking status, dietary choices and frequency of physical activity.

The cancer nurses’ nutrition promotion practices were examined with single choice and multiple-choice questions. Items sought to assess nurses’ opinions on responsibility of healthy eating promotion in their hospital and whether they felt the dietician/nutritionist, oncologist, themselves, or others were the primary person in charge. Attitudes towards healthy eating promotion during different stages of cancer treatment (pre-, during- and post-treatment) were investigated with multiple-choice items.

Cancer nurses’ beliefs on beneficial effects of healthy eating were assessed on a Likert scale ranging from 1 (strongly disagree) to 4 (strongly agree) on seven different factors: (1) improves health related QoL, (2) improves weight management, (3) improves mental health, (4) improves activities of daily living, (5) reduces risk of cancer recurrence, (6) reduces risk of other diseases and (7) reduces tumour specific comorbidities. Furthermore, cancer nurses were asked their opinion on whether they thought their patients are generally uninterested in healthy eating, that promoting healthy eating is entirely up to them (i.e., responsibility of the nurse), if they believe they should promote healthy eating and if there is a strong evidence base to promote healthy eating.

Commonly cited barriers to healthy behaviour promotion were also included in the survey items. These barriers included lack of time, lack of adequate support structures, lack of expertise, lack of knowledge, risk to survivor, perceived scope of practice (“not my job”) and finally not having any barriers for healthy eating promotion (Blake & Patterson, 2015; Brandes et al., 2015; Brotons et al., 2005). The respondents were asked to rank each barrier according to their personal experience from primary barrier (3 points), secondary barrier (2 points) to tertiary barrier (1 point).

### Statistical analyses

Data were evaluated using SPSS (version 22.0; IBM Corp., Armonk, NY, US) and Excel 2010 (Microsoft Corp., US) software. Demographics, nutrition promotion practices and beliefs of cancer nurses were analysed using descriptive statistics. We chose to do sub-group comparisons across hospital location (metropolitan vs. regional and rural) and years of practice (<25 years and ≥ 25 years) due to comparable sample sizes of these sub-groups. Sub-group comparisons based on cancer nurse gender (male or female), hospital type (private or public) and cancer group specialization were not performed as the sample sizes of these sub-groups were too unbalanced. Selected sub-group comparisons were examined using one way analyses of variance (ANOVA) for continuous variables or chi-square tests for categorical variables related to the nurses’ healthy eating promotion practices, beliefs and barriers. Descriptive data was presented as mean and standard deviation or counts and frequencies depending on the type of data. All statistical tests were two-sided with a significance level of *p* < 0.05.

## Results

All registered members with a valid email address of the CNSA and NZNO received an invitation to complete the online questionnaire between October or December 2013 and July 2014. The exact response rate is unknown; however, as a guide the number of members at that time ranged from 500 to 1,500 in each organization. A total of 123 registered nurses from Australia and New Zealand completed the online questionnaire.

Details of the demographic profiles are presented in Table 1. In summary, the majority of participants (95.9%) were female, the mean age was 48.7 ± 10.5 years and the mean number of years in practice was 23.0 ± 11.7 years. The most common field nurses worked in was general oncology (*n* = 48, 40%), with a specialisation in gynaecological cancers being the second most common group (*n* = 21, 17.5%). The vast majority of cancer nurses were based in public (*n* = 102, 84%) rather than private (*n* = 19, 16%) hospitals. The location of the hospitals was distributed almost equally with 51.7% working in the metropolitan area and 48.3% in regional or rural hospital locations.

**Table 1:** Sample demographics.

Sample (*n* = 123)	*n* (%)
**Age** (**years**, ***n = 121***)	
<25	4 (3.3)
26–35	10 (8.2)
36–45	31 (25.6)
46–55	40 (33.1)
56–65	33 (27.3)
>65	3 (2.5)
**Gender** (**n = 122**)	
Male	5 (4.1)
Female	117 (95.9)
**Highest educational qualification** (**n = 123**)	
Registered nurse/bachelor’s degree	34 (27.6)
Diploma/graduate certificate	55 (44.7)
Master’s degree	33 (26.8)
**Cancer group specialisation** (**n = 120**)	
General oncology	48 (40)
Gynaecological (breast, ovary)	21 (17.5)
Haematology	9 (7.5)
Urogenital (prostate, bladder)	7 (5.8)
Palliative care settings	7 (5.8)
Lung	6 (5)
Gastrointestinal/colorectal	6 (5)
Other (head and neck cancer, sarcoma, skin lymphoma, paediatrics)	11 (9.2)
**Years practicing** (**years**, **n = 121**)	
<5	8 (6.6)
5–14.9	27 (22.3)
15–24.9	27 (22.3)
≥25	59 (48.8)
**Hospital** (**n = 121)**	
Public	102 (84.3)
Private	19 (15.7)
**Location** (**n = 120**)	
Metropolitan	62 (51.7)
Regional	39 (32.5)
Rural	19 (15.8)
**Regular reader of professional journals** (**n = 123)**	
Yes	60 (48.8)
No	63 (51.2)

Considering their own lifestyle behaviours, the majority of the respondents reported that they followed healthy eating guidelines on a regular basis (88%), and were not currently smoking (98%). Almost half (47%) of the sample described themselves as physically active in that they performed at least 5 sessions of moderate intensity exercise for 30 min or more per week.

The current nutrition promotion practices of cancer nurses are summarised in Table 2. Most cancer nurses considered the dietician/nutritionist (35.9%) the primary person responsible for providing healthy eating advice to patients. However, 32.5% of nurses considered themselves the primary person responsible for addressing their patients’ nutrition concerns. Almost 75% of the respondents stated that the most common time they promoted healthy eating was during cancer treatment. More than half of nurses (52.8%) promoted healthy eating to their patients during all cancer stages (pre-, during, and post-treatment).

**Table 2:** Nutrition promotion practices and sub-group comparison for years of practice and hospital location.

	*n* = 123^a^ (%)	Years of practice *n* = 121 (%)			Hospital location *n* = 120 (%)		
		<25 years *n* = 60 (49.6)	≥25 years *n* = 61 (50.4)	*p*-value	Metro *n* = 62 (51.7)	Rural & regional *n* = 58 (48.3)	*p*-value
**In your opinion, who is the primary person responsible for healthy eating in your hospital?**							
Me	40 (32.5)	17 (28.3)	23 (37.7)	0.273	17 (27.4)	23 (39.7)	0.171
Nutritionist/dietician	43 (35.0)	25 (41.7)	17 (27.9)	0.111	29 (46.8)	14 (24.1)	0.015^*^
Oncologist	4 (3.3)	3 (5.0)	1 (1.6)	0.301	0 (0)	4 (6.9)	0.099
Other	20 (16.3)	12 (20.0)	8 (13.1)	0.308	10 (16.1)	9 (15.5)	0.717
I don’t know	6 (4.9)	2 (3.3)	4 (6.6)	0.414	4 (6.5)	2 (3.4)	0.691
**Please indicate in which stage of cancer treatment healthy eating is promoted** ^b^							
Pre treatment	77 (62.6)	40 (66.7)	37 (60.7)	0.492	37 (59.7)	40 (69.0)	0.289
During treatment	92 (74.8)	49 (79.0)	43 (70.5)	0.150	46 (74.2)	45 (77.6)	0.664
Post treatment	79 (64.2)	43 (69.4)	36 (59.0)	0.144	40 (64.5)	39 (67.2)	0.753
Every stage	65 (52.8)	34 (54.8)	31 (50.8)	0.519	32 (51.6)	33 (56.9)	0.562
I don’t know	10 (8.1)	4 (6.5)	6 (9.8)	0.527	7 (11.3)	3 (5.2)	0.226

**Notes.**

a Numbers may not equal 123 due to missing data or missing response.

b Multiple-choice answers were possible.

Metro Metropolitan

* *p* < 0.05 group differences based on Pearson Chi-squared analysis.

Sub-group comparisons were analysed for years of practice (<25 years vs. ≥25 years) as well as hospital location (metropolitan versus rural and regional located hospitals). No significant differences in nutrition practices were observed between nurses with more or less than 25 years of experience. Significantly more nurses working in metropolitan areas considered the nutritionist/dietician as the primary person responsible for healthy diet advice compared with nurses in rural hospitals (46.8% vs 24.1%, *p* = 0.015, respectively).

The current nutrition promotion beliefs are summarised in Table 3. Most cancer nurses agreed or strongly agreed that healthy eating improved health-related QoL (85.4%), weight management (82.9%), mental health (80.5%), activities of daily living (79.7%) and reduces the risk of other chronic diseases (79.7%) for cancer patients. Moreover, 70.7% agreed or strongly agreed that healthy eating could reduce risk of cancer recurrence and 63.4% believed that healthy eating could reduce tumour specific comorbidities. While 68.3% of cancer nurses believed that healthy eating had some benefits for their patients, 29.3% did not respond to this question.

**Table 3:** Current beliefs of cancer nursers regarding healthy eating for cancer patients.

What benefits may healthy eating have for your cancer patients?^a^*n* = 123^b^	Strongly agree *n* (%)	Agree *n* (%)	Disagree *n* (%)	Strongly disagree *n* (%)	No response *n* (%)
Improve health related quality of life	62 (50.4)	43 (35.0)	2 (1.6)	0	16 (13.0)
Improve weight management	64 (52.0)	38 (30.9)	3 (2.4)	0	18 (14.6)
Improve mental health	51 (41.5)	48 (39.0)	6 (4.9)	0	18 (14.6)
Improve activities of daily living	48 (39.0)	50 (40.7)	6 (4.9)	0	19 (15.4)
Reduce risk of cancer recurrence	31 (25.2)	56 (45.5)	15 (12.2)	1 (0.8)	20 (16.4)
Reduce risk of other chronic diseases	44 (35.8)	54 (43.9)	4 (3.3)	0	21 (17.1)
Reduce tumour specific comorbidities	25 (20.3)	53 (43.1)	20 (16.3)	2 (1.6)	23 (18.7)
No benefits	0	3 (2.4)	9 (7.3)	75 (61.0)	36 (29.3)
My cancer patients are generally uninterested in healthy eating	4 (3.3)	13 (10.6)	73 (59.3)	13 (10.6)	20 (16.3)
Whether or not I promote healthy eating to my cancer patients is entirely up to me	18 (14.6)	42 (34.1)	25 (20.3)	13 (10.6)	25 (20.3)
My fellow nurses believe I should be promoting healthy eating to my cancer patients	14 (11.4)	59 (48.0)	18 (14.6)	4 (3.3)	28 (22.8)
There is a strong evidence base suggesting I should promote healthy eating to my cancer patients	41 (33.3)	52 (42.3)	3 (2.4)	1 (0.8)	26 (21.1)

**Notes.**

a All questions rated on a 4-point Likert scale with 1, strongly disagree; 2, disagree, 3, agree and 4, strongly agree. Metro, Metropolitan.

b Numbers may not equal 123 due to missing data.

A range of other beliefs may affect the cancer nurses’ promotion of healthy eating to their patients. For example, 75.5% of the cancer nurses believed the evidence base for healthy eating promotion to their patients was strong, and 69.9% of the nurses felt that their cancer patients were interested in healthy eating advice. More than half (59.4%) felt that their cancer nurse colleagues believed that all nurses should promote healthy eating to cancer patients. Interestingly, only 49% of the nurses felt that whether or not they promoted healthy eating to their patients was entirely their own decision, and that such a decision was not affected by their work environment or primary scope of practice.

Table 4 compares the current healthy eating beliefs of the cancer nurses across years of practice and hospital location (metropolitan versus rural and regional). In general, there were few significant effects for years of practice or hospital location on the cancer nurses’ healthy eating beliefs. The exceptions to this were that less experienced cancer nurses (<25 years of practice) were significantly more likely to believe that healthy eating could reduce tumour specific comorbidities than their more experienced counterparts (*p* = 0.042). The cancer nurses working in metropolitan hospitals were also significantly more likely to believe healthy eating could have positive impacts on health related QoL (*p* = 0.046).

**Table 4:** Comparison of cancer nurses’ attitudes towards healthy eating across sample demographics.

What benefits may healthy eating have for your cancer patients?^a^	Years of practice			Location		
	<25 years	>25 years	*p*	Metro	Rural & **regional**	*p*
Improve health related quality of life	3.2 ± 1.1	2.9 ± 1.4	0.254	3.3 ± 1.1	2.9 ± 1.3	0.046^*^
Improve weight management	3.2 ± 1.1	2.9 ± 1.4	0.200	3.2 ± 1.1	2.9 ± 1.4	0.158
Improve mental health	3.1 ± 1.1	2.7 ± 1.4	0.149	3.1 ± 1.1	2.8 ± 1.4	0.231
Improve activities of daily living	2.9 ± 1.2	2.8 ± 1.4	0.547	3.1 ± 1.0	2.7 ± 1.5	0.140
Reduce risk of cancer recurrence	2.8 ± 1.1	2.5 ± 1.4	0.270	2.8 ± 1.1	2.5 ± 1.3	0.173
Reduce risk of other chronic diseases	3.0 ± 1.2	2.6 ± 1.4	0.159	3.0 ± 1.2	2.6 ± 1.4	0.111
Reduce tumour specific comorbidities	2.7 ± 1.1	2.2 ± 1.4	0.042^*^	2.6 ± 1.3	2.3 ± 1.3	0.169
No benefits	0.9 ± 0.6	0.7 ± 0.6	0.135	0.9 ± 0.6	0.7 ± 0.6	0.184
My cancer patients are generally uninterested in healthy eating	1.8 ± 0.8	1.6 ± 1.0	0.257	1.8 ± 0.9	1.6 ± 0.9	0.248
Whether or not I promote healthy eating to my cancer patients is entirely up to me	2.1 ± 1.3	2.1 ± 1.4	0.849	2.2 ± 1.3	2.0 ± 1.3	0.401
My fellow nurses believe I should be promoting healthy eating to my cancer patients	2.2 ± 1.2	2.2 ± 1.4	0.882	2.2 ± 1.2	2.2 ± 1.4	0.946
There is a strong evidence based suggesting I should promote healthy eating to my cancer patients	2.6 ± 1.4	2.7 ± 1.4	0.744	2.7 ± 1.4	2.6 ± 1.4	0.842

**Notes.**

Data presented as mean ± SD.

a all items rated on 4-point Likert scale, with 1, strongly disagree; 2, disagree; 3, agree and 4, strongly agree. Metro, Metropolitan

* *p* < 0.05, group differences based on one way analysis of variance (ANOVA).

Table 5 provides data on the most frequently cited barriers to healthy eating promotion. The most commonly cited barriers for not promoting healthy eating were lack of time (25.8%), lack of adequate support structures (17.3%), lack of expertise (12.2%), risk to cancer patient (5.1%) and lack of knowledge (4.4%); with 2.2% not considering it their job to give dietary advice. However, almost a third (31.6%) reported no barriers in promoting a healthy diet. Sub-group analysis between nurses working in metropolitan vs. regional/rural hospitals or with more or less than 25 years of experience revealed some significant differences in the frequency of most commonly cited barriers to healthy eating promotion. Compared to the less experienced cancer nurses (<25 years of practice), those with ≥25 years of experience were more likely to state they had no barriers in promoting healthy eating (*p* = 0.045). The less experienced nurses also cited a lack of knowledge significantly more often as a perceived barrier to healthy eating promotion than their more experienced counterparts (*p* < 0.001). Regarding hospital location, cancer nurses working in regional and rural hospitals were more likely to cite a risk to the cancer patient as a barrier to healthy eating promotion than those working in metropolitan hospitals (*p* < 0.001).

**Table 5:** The most frequently cited nutrition promotion barriers^a^.

*N* = 123	I do not have barriers in promoting healthy eating	Lack of time	Lack of adequate support structures	Lack of expertise	Risk to patient	Lack of knowledge	Not my job
**Total *n*** ^b^ **(%)**	142 (31.6)	116 (25.8)	78 (17.3)	55 (12.2)	23 (5.1)	20 (4.4)	10 (2.2)
**Location**							
Metropolitan	73 (30.8)	63 (26.6)	49 (20.7)	30 (12.7)	4 (1.7)	11 (4.6)	5 (2.1)
Rural & regional	69 (33.2)	53 (25.5)	29 (13.9)	22 (10.6)	20 (9.6)	7 (3.4)	4 (1.9)
*p* value	0.658	0.820	0.088	0.520	0.0004^**^	0.501	0.890
**Years of practice**							
<25 years	63 (26.7)	59 (25.0)	39 (16.5)	36 (15.3)	9 (3.8)	19 (8.1)	6 (2.5)
>25 years	79 (37.4)	54 (25.5)	39 (18.5)	19 (9.0)	14 (6.6)	1 (0.5)	4 (1.9)
*p* value	0.045^*^	0.901	0.621	0.058	0.192	0.0001^**^	0.646

**Notes.**

a Points given on 3-point rating scale: highest rated barrier 3 points, lowest rated barrier 1 point.

b Numbers may not equal 123 due to missing data among groups.

* *p* < 0.05.

** *p* < 0.01, group differences based on Chi-squared analysis.

## Discussion

As emerging research indicates the benefits of maintaining a healthy diet in cancer patients and survivors (Langius et al., 2013; Millar & Davison, 2012; Mokdad et al., 2003), this study sought to gain insight into current nutrition promotion practices, beliefs and perceived barriers of cancer nurses in Australia and New Zealand. Such insight is important as: (1) no peer reviewed research on this topic has been published in Australasia; and (2) cancer nurses often have more interaction with cancer patients and therefore more opportunities to discuss healthy behaviours such as nutrition than oncologists or general practitioners (Blake & Patterson, 2015; Karvinen et al., 2012; O’Hanlon & Kennedy, 2014).

Our results demonstrate that while the cancer nurses believed that providing healthy eating advice to cancer patients was primarily the responsibility of dieticians, they also felt that nurses played a very important role in providing general healthy eating advice to their patients. Cancer nurses’ views on healthy eating promotion appear consistent with other studies, whereby nurses provide important information to cancer patients on topics as broad as sexual health (Kotronoulas, Papadopoulou & Patiraki, 2009) and the benefits of physical activity (Karvinen et al., 2012). This would suggest that the promotion of general healthy eating guidelines such as increasing fruit and vegetable intake and reducing refined grains, processed meats and sugar intake by cancer nurses to their patients is not beyond their current scope of practice.

The nurses in the current study felt that oncologists were the least likely health professional group (3.3%) to be the primary provider of nutritional advice to patients. Such results appear consistent with the relatively low proportion of oncologists providing lifestyle advice, with only 28% actually recommending physical activity to any survivor at the time of consultation (Jones et al., 2005a). The relatively minor role that oncologists appear to play in promoting healthy behaviours such as healthy eating and physical activity to their patients may reflect a number of factors. Most notably, the primary role of oncologists and other physicians is to give their patients accurate information about treatment options and to discuss medical issues including biological test results and treatment options (Leahy et al., 2012). Due to their relative lack of training in nutrition and physical activity and time constraints during consultations, they may briefly mention the importance of healthy behaviours to patients, but leave more in-depth discussions to other health professionals, such as nurses. The cancer nurses may, therefore, be able to reinforce health promotion messages initially delivered by the oncologist, assisting the patients change their behaviour (Van der Molen, 1999).

Our results indicated that the majority of cancer nurses are promoting healthy eating to their patients prior to (62.6%), during (74.8%) and post-treatment (64.2%), with 52.8% promoting healthy eating at every stage of the treatment process. The promotion of healthy diet across all treatment phases is important as healthy eating has numerous benefits for cancer patients, with some of these differences perhaps more important at the various treatment stages. Specifically, patients may experience alterations in appetite and require somewhat different nutritional intakes over each treatment phase in order to maintain sufficient nutritional intakes, body composition and QoL (Aapro et al., 2014; Hung et al., 2013; Rock et al., 2012).

The relatively high rate of healthy eating promotion by cancer nurses in this study appears consistent with their beliefs around the importance of healthy eating for their patients. Collectively these results indicate that while cancer nurses believe that there is considerable evidence that healthy eating has many benefits for their cancer patients, they are not completely sure about the strength or extent of this evidence. This may reflect the relatively limited number of studies examining specific nutritional interventions within each cancer type, different treatments options or at different treatment phases. Nevertheless, healthy eating and other healthy behaviours may help to reduce risk of the long-term and late effects of cancer treatment including diabetes and obesity (Mokdad et al., 2003), osteoporosis (Millar & Davison, 2012) and overall QoL (Langius et al., 2013).

Interestingly, cancer nurses thought that a high proportion (69.9%) of their patients were interested in healthy eating. This perception is inconsistent with recent data from the UK, where one of the most consistent barriers cited by nurses, surgeons, and physicians to providing lifestyle advice, was lack of cancer patient interest (Williams et al., 2015). Other data suggest that cancer patients would welcome advice on health promotion and lifestyle (Anderson, Steele & Coyle, 2013; Demark-Wahnefried et al., 2000; Keogh et al., 2014). These discrepancies throughout the literature suggest that improved communication is necessary to meet patients’ needs. Nurses interested in improving their healthy eating (or other healthy behaviours) communication and promotion should consider the integration of evidence-based practice, with behaviour change theories such as the SCT shown to be an effective approach in delivering such information to cancer patients (Stacey et al., 2015).

The current study was also interested in identifying what barriers the nurses may have in promoting healthy eating to their cancer patients. Our findings demonstrated that lack of time, adequate support structures and nutritional expertise were the major perceived barriers, and therefore appear consistent with research involving other health professionals in promoting healthy behaviours to patients (Blake & Patterson, 2015; Brandes et al., 2015; Brotons et al., 2005). These barriers also appeared similar to the views of 236 cancer patients regarding consultations with their health professionals, with a lack of consultation time and an inability of the health professionals to provide accurate information cited as some of the major issues (Brandes et al., 2015). To overcome these issues and improve patient outcomes, more effort should be placed upon providing continuing education for cancer nurses around the benefits of healthy eating and the provision of greater support structures, such as referral pathways or specific nutritional resources to provide to their patients. Increasing consultation times between the patient and the oncology care team could also lead to a better communication however is not realistic or practical to expect nurses to spend more time with their patients in most healthcare institutions. Hospital and national healthcare policies may therefore, need to be considered to reduce some of these barriers to healthy eating promotion by the cancer nurses.

It was noteworthy that the years of practice (experience of the nurses) or location of the hospital in which the cancer nurses worked resulted in few significant differences in their nutritional promotion practices, beliefs, or barriers. This finding was somewhat unexpected as it was thought that more experienced nurses may be more likely to promote healthy behaviours like healthy eating than less experienced nurses. This could be due to the fact that more experienced nurses often accompany their cancer patients a long time throughout different treatment stages therefor have the desire to minimize patients’ distress as reported in former studies (Miles, Simon & Wardle, 2010). We also expected that nurses in metropolitan hospitals would likely have greater access to specialised service providers such as dieticians than nurses working in regional and rural hospitals. On this basis, it was expected that the metropolitan nurses may be more reluctant to work outside their primary area of expertise and be less involved in promoting healthy behaviours such as healthy diet to their cancer patients. The relative lack of effect of years of practice and hospital location on the cancer nurses’ healthy diet promotion practices, beliefs and barriers is a positive finding that increases the generalisability of these results and highlights the strong interest cancer nurses have in providing the best care to their cancer patients.

This study is not without its limitations. The sample size of 123 nurses who completed the survey only represents a small proportion of the registered Australasian cancer nurses. Therefore, the sample recruited in the study may not be truly representative of Australasian cancer nurses, especially those working in private hospitals, as only 19 of the 123 respondents currently work in this sector. Nevertheless, the sample size of the current study is greater than some studies (O’Hanlon & Kennedy, 2014; Spellman, Craike & Livingston, 2014) or similar to other quantitative survey-based studies examining healthy behaviour promotion by health professionals to cancer patients (Daley et al., 2008; Karvinen et al., 2010). Further, it was not known if the cancer nurses who responded to the survey had access to a dietician or nutritionist within their institution and if the presence or absence of such trained dietary professionals would have influenced their healthy eating beliefs and promotion practices to their patients.

The results of this study add to the existing literature regarding the promotion of healthy eating by health professionals to their cancer patients, particularly cancer nurses working in Australasia. Specifically, there is very limited research about healthy behaviour promotion (in general) of health professionals to cancer patients in Australasia (Spellman, Craike & Livingston, 2014), or international research on the role of cancer nurses in healthy behaviour promotion (Karvinen et al., 2012; Williams, Beeken & Wardle, 2013). It is hoped the results of this study will encourage additional research into the current healthy behaviour promotion practices of cancer nurses, while also highlighting some of the barriers they face in providing this important information to their patients. Based on current evidence (Langius et al., 2013; Millar & Davison, 2012; Mokdad et al., 2003), it would appear likely that an increased promotion of healthy behaviours, including a healthy diet and physical activity by health professionals to cancer patients would result in improved survivor outcomes. Cancer nurses are ideally placed to deliver these initial messages and to refer interested patients to dieticians or nutritionists for further healthy eating assistance.

## Supplemental Information

10.7717/peerj.1396/supp-1 Supplemental Information 1 Raw data
